# Identification of druggable inhibitory immune checkpoints on Natural Killer cells in COVID-19

**DOI:** 10.1038/s41423-020-0493-9

**Published:** 2020-07-01

**Authors:** Olivier Demaria, Julien Carvelli, Luciana Batista, Marie-Laure Thibult, Ariane Morel, Pascale André, Yannis Morel, Frederic Vély, Eric Vivier

**Affiliations:** 1 Innate Pharma, 117 avenue de Luminy, Marseille, 13009 France; 2 Assistance Publique des Hôpitaux de Marseille, Hôpital de la Timone, Réanimation des Urgences, Marseille, 13005 France; 3 Aix Marseille Université, CNRS, INSERM, Centre d’Immunologie de Marseille-Luminy, Marseille, 13009 France; 4 Assistance Publique des Hôpitaux de Marseille, Hôpital de la Timone, Immunology, Marseille Immunopole, Marseille, 13005 France

**Keywords:** Viral infection, Drug discovery

Infection with SARS-COV-2 is the cause of COVID-19 and has generated an unprecedented health crisis worldwide. While most of the patients experience mild symptoms, around 20% develop severe disease, characterized by pneumonia and in the worst cases by acute respiratory distress syndrome (ARDS).^1^ The analysis and understanding of the immune responses arising in the course of SARS-COV-2 infection may help to propose therapeutic solutions. Due to the crucial role of Natural Killer (NK) cells in antiviral immune responses,^2^ we analyzed NK cells in blood from a cohort of 82 individuals: 10 healthy controls (HC), 10 paucisymptomatic COVID-19 patients (pauci), 34 patients with pneumonia (pneumo) and 28 patients with ARDS due to SARS-CoV-2 infection. The absolute numbers of peripheral blood NK cells, B, CD4^+^, and CD8^+^ T lymphocytes were lower in the pneumonia and ARDS groups than in healthy controls, consistent with previously published results^3^ (Fig. 1a). We investigated the NK cell subsets further and found that among CD45^+^CD3^−^CD56^+^ total NK cells the proportion of mature NK cells, a subset defined on the basis of its expression of the CD16 and CD57 cell surface receptors, was markedly lower in patients with ARDS (Fig. 1b). Given their role in viral infection, the loss of mature NK cells may contribute to the pulmonary complications occurring in the most severe cases of COVID-19. We then focused our analysis on molecular pathways likely to improve NK cell antiviral activity to promote SARS-CoV-2 clearance, and analyzed the expression of several immune checkpoints. Given the availability of therapeutic monoclonal antibodies blocking the immunosuppressive functions of PD-1,^4^ NKG2A,^5^ and CD39^6^ initially developed for cancer therapies, we analyzed the expression of these molecules on NK cells in our cohort. PD-1 and NKG2A are cell surface receptors, and their engagement with their ligands, PD-L1 and HLA-E, respectively, inhibits the function of T and NK cells. CD39 is an ectoenzyme that cleaves extracellular ATP and ADP, which can be released from dead cells upon viral infection, leading to the generation of adenosine, which has strong immunosuppressive effects on T and NK cells.

**Fig. 1:**
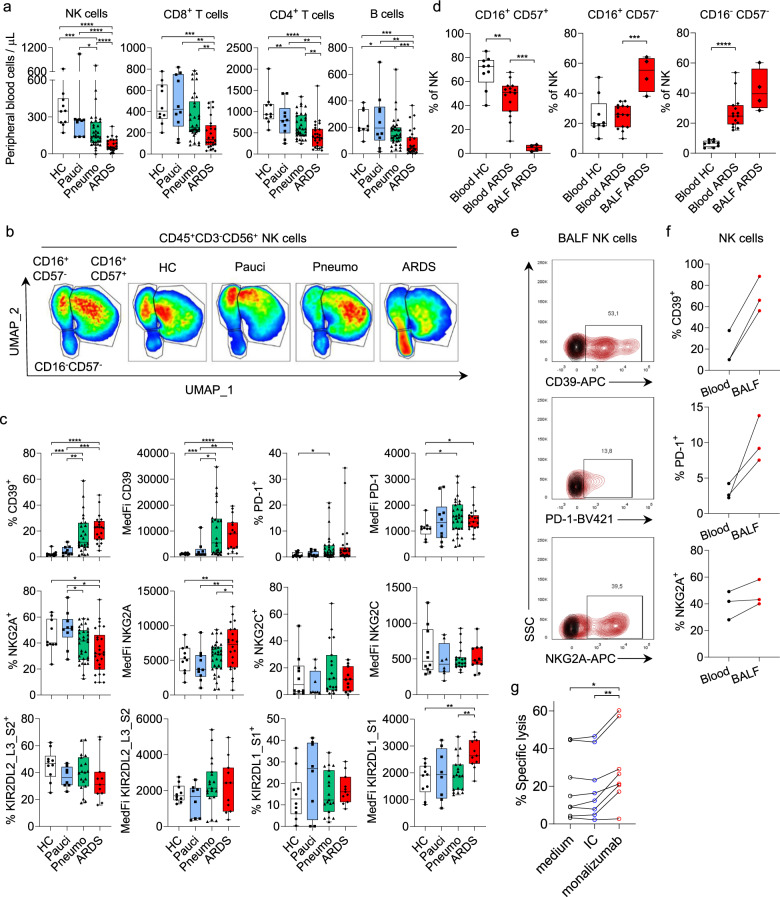
COVID-19 is associated with lymphopenia and a dysfunctional NK cell phenotype. **a** Absolute numbers of circulating NK, B, CD4^+^ and CD8^+^ T cells per microliter of peripheral blood from healthy donors and COVID-19 patients. **b** Left: UMAP projection of concatenated peripheral CD45^+^CD3^−^CD56^+^ NK cells from all samples identifies 3 NK clusters: CD16^-^CD57^−^, CD16^+^CD57^−^ and CD16^+^CD57^+^. Right: UMAP projections of concatenated peripheral blood NK cells from healthy donors and COVID-19 patients. Red indicates the highest cell density. **c** Phenotyping of NK cells in peripheral blood from healthy donors and COVID-19 patients. The percentages of total NK cells and MedFI for marker-positive NK cells are shown. **d** Percentages of CD16^+^CD57^+^, CD16^+^CD57^−^, and CD16^−^CD57^−^ subsets in CD45^+^CD3^−^CD56^+^ NK cells in the blood of HCs (white) and ARDS patients  (red) and BALF from ARDS patients (red). **e** Representative flow cytometry contour plots from three ARDS patients showing CD39, PD-1, and NKG2A expression (red) vs. fluorescence minus one (FMO, black) on NK cells from BALF. **f** Percentages of CD39^+^, PD-1^+^ and NKG2A^+^ NK cells, cells in blood (black) and BALF (red) from the same ARDS patients. **g** Specific K562-HLA-E lysis by NK cells isolated from ARDS PBMCs incubated with medium (black), isotype control (IC, blue) or monalizumab (red). **a-g**, Each symbol represents a single donor. Experimental conditions and statistics details are provided in supplementary material.

We observed the presence of a CD39-expressing NK cell population in the blood of the COVID-19 patients of the pneumonia and ARDS groups that was absent in the HC and paucisymptomatic groups. Moreover, the cell surface density of CD39, which is known to be upregulated by hypoxia, was much higher in this subset than observed in normal conditions, as shown by the small number of CD39-expressing NK cells from HC (Fig. 1c). IL-6 induces CD39 expression on tumor infiltrating NK cells and might participate to their impaired functions in the context of cancer.^7^ Expression of CD39 on NK cells from COVID-19 patients may thus be explained by the levels of circulating IL-6 that rise with disease severity. Similarly, the PD-1 receptor was upregulated on NK lymphocytes in COVID-19 patients, and several pneumonia and ARDS COVID-19 patients had a particularly large subset of NK cells expressing PD-1. The expression of PD-1 on NK cells has been described in some human cancers, but a high frequency of PD-1^+^ NK cells, as observed in the peripheral blood of some severely affected COVID-19 patients, is uncommon. We found that the NKG2A-expressing NK cell subset was smaller in patients with ARDS, although the cell surface density of NKG2A was upregulated in this group (Fig. 1c). By contrast, the expression of NKG2C, an activating receptor of HLA-E implicated in the control of CMV infections, remained unmodified in COVID-19 patients (Fig. 1c). Among the inhibitory or activating receptors for HLA-C, the expression of KIR2DL1/S1 was also stronger on the NK cells of ARDS patients, whereas KIR2DL2/L3/S2 levels remained unmodified (Fig. 1c). Thus, circulating NK cells in COVID-19 patients display an upregulation of the inhibitory receptor NKG2A that is associated with disease severity, consistent with recent results.^8^ Bronchoalveolar lavage fluid (BALF) analysis showed a lack of CD16^+^CD57^+^ mature NK cells in the lungs of ARDS patients (Fig. 1d), suggesting that the decrease in mature NK cell levels observed in blood (Fig. 1b, d) is not a consequence of their migration to infected lungs. In addition, high levels of CD39, PD-1, and NKG2A expression were also observed in NK cells isolated from the BALF of ARDS COVID-19 patients, and these levels were even higher than those observed on blood cells of the patients tested (Fig. 1e, f). Together, these results show that NK cells in the blood and BALF of COVID-19 patients display signs of dysfunction resembling those described in cancers, at least to some extent. The blockade of CD39, PD-1, or NKG2A has been shown to harness NK cell immunity in cancers and would be of interest in COVID-19, to enhance NK cell-mediated viral clearance. We therefore analyzed whether NK cells from COVID-19 patients remained functional and could be reinvigorated. We found that NK cells isolated from the blood of ARDS COVID-19 patients retained cytotoxic functions, and that incubation with monalizumab,^5^ an anti-NKG2A mAb blocking the inhibitory interaction with HLA-E, was able to unleash their killing ability (Fig. 1g). These data suggest that therapeutic interventions may improve NK cell functions, facilitating virus elimination, and support the repurposing of therapeutic mAbs targeting inhibitory molecules in COVID-19. The immune system plays an important role in virus elimination in COVID-19, but it is becoming clear that the infiltration and activation of myeloid cells in infected lungs are also critical for ARDS development.^9^ Highly activated NK cells have been shown to worsen lung injury in respiratory infection models, and the use of therapeutic tools to improve NK cell functions should, thus, be carefully considered in severe cases of COVID-19.^10^ However, our data, showing a decrease in NK cell numbers and a dysfunctional state of these cells in the blood and lungs of COVID-19 patients, suggest that NK cells do not participate in the exaggerated inflammatory response observed in ARDS. Thus, therapies targeting PD-(L)1, NKG2A and CD39 should be investigated as means of boosting NK cell antiviral immunity in patients at early stages of SARS-CoV-2 infection.

## Supplementary information

Supplementary material
