# Supplementary material for: Identification of druggable inhibitory immune checkpoints on Natural Killer cells in COVID-19
Source: Cell Mol Immunol. 2020 Jul 1;17(9):995–7. doi: 10.1038/s41423-020-0493-9 (PMC7327215; doi:10.1038/s41423-020-0493-9)
Supplement: Supplementary file 1 — Supplementary material [file 41423_2020_493_MOESM1_ESM.docx]

**Supplementary materials**

**Methods**

**Reagent table**

| **Reagent** | **Reference** | **Provider** |
| --- | --- | --- |
| DPBS (1X) | 14190-094 | Gibco |
| RPMI medium 1640 (1X) | 31870-025 | Gibco |
| Sodium pyruvate 100 mM (100X) | 11360-039 | Gibco |
| L-glutamine 200 mM (100X) | 25030-024 | Gibco |
| Minimum essential medium non-essential amino acids solution | 11140-035 | Gibco |
| Trypan blue stain (0.4%) | 15250-061 | Gibco |
| Ficoll-Paque PLUS | 17-1440-03 | GE Healthcare |
| Fetal bovine serum | F7524 | Sigma |
| Dimethyl sulfoxide | D2650-100ML | Sigma |
| CD57-FITC clone NK-1 | 555619 | BD Biosciences |
| CD33- PECF594 clone WM53 | 562492 | BD Biosciences |
| CD19-PECy7 clone SJ25C1 | 557835 | BD Biosciences |
| CD279-BV421 clone EH12.2H7 | 329920 | BioLegend |
| CD15-BV510 clone W6D3 | 563141 | BD Biosciences |
| CD45-BV711 clone HI30 | 564357 | BD Biosciences |
| CD56-BV786 clone NCAM16.2 | 564058 | BD Biosciences |
| CD16-BUV395 clone 3G8 | 563785 | BD Biosciences |
| CD3-BUV496 clone UCHT1 | 564809/612940 | BD Biosciences |
| CD14-BUV737 clone M5E2 | 564444/612763 | BD Biosciences |
| HLA-DR-AF700 clone L243 | 307626 | BioLegend |
| LIVE DEAD NEAR IR | L34976 | ThermoFisher |
| Mouse serum | 015-000-120 | Jackson ImmunoResearch |
| NKG2A APC clone REA110 | 130-113-563 | Miltenyi Biotec |
| CD39 APC clone A1 | 328210 | BioLegend |
| NKG2C PE clone 134591 | FAB138P-100 | R&D Systems |
| CD158b1/b2,j (KIR2DL2/L3/S2) PE clone GL183 | IM2278U | BC |
| CD158a/h (KIR2DL1/S1) APC clone 11PB6 | 130-092-685 | Miltenyi Biotec |
| CD45 FITC clone HI30 | 304038 | BioLegend |
| CD16 FITC | 556616 | BD Biosciences |
| 6-Color TBNK Reagent with Trucount Tubes | 662995 | BD Biosciences |
| EDTA | 15575-038 | Ivitrogen, |
| Sodium azide | 71290-100g | Sigma |
| Optilyse C solution | A11895 | Beckman Coulter |
| CytoFix | 554655 | BD Biosciences |
| NK Cell Isolation Kit, human | 130-092-657 | Miltenyi Biotec |
| Triton X-100 solution | 93443-100ML | Sigma |

**Ethics approval statement**

All the patients (and/or initially their family) provided written informed consent before sampling and for the use of their clinical and biological data. The study protocol was approved on 03-27-2020 by the Committee for the Protection of Persons Ile-de-France III – France (#2020-A00757-32).

**Sample collection**

Samples of patients infected with SARS-CoV-2 and healthy controls were collected from three hospitals (Timone and Nord University Hospitals and Laveran Military Hospital, Marseille). Study subjects, design and clinical considerations have been previously described^1^. Flow cytometry analyses were performed on fresh blood samples (EDTA tubes) and BALFs, immediately after collection. Whole blood collected in EDTA tubes and diluted 1/2 in PBS was used to isolate PBMCs by centrifugation on a Ficoll gradient. PBMCs were collected and 10^7^ PBMCs per vial were frozen in freezing medium (90% FCS + 10% DMSO).

^1^ Carvelli, J et al. Identification of immune checkpoints in COVID-19, 07 May 2020, PREPRINT (Version1 available at Research Square [+https://doi.org/10.21203/rs.3.rs-27340/v1+]

**Flow cytometry**

Blood collected into EDTA tubes was washed in PBS before staining with LiveDead (Thermo Fisher) according to the manufacturer’s instructions. Cells were incubated with mouse serum to saturate the Fc receptors, and were then incubated in the appropriate antibody cocktail. Red blood cells were lysed in Optilyse C Solution (Beckman Coulter), according to the manufacturer’s instructions. Cells were fixed in Cell Fix solution (BD), according to the manufacturer’s instructions. Data were acquired in an LSRFortessaX20 flow cytometer. The FCS3.0 files obtained were exported from BD FACSDiva software and imported into FlowJo v.10.5.2 (BD Biosciences). Automated compensation was calculated by FACSDiva software with single-stained compensation beads. This compensation matrix was analyzed in detail in FlowJo, by investigating the N-by-N view feature and the pairwise expression of all proteins stained in this study. Fluorescence minus one (FMO) experiments were run before this study, to facilitate optimization of the compensation matrix. We then adjusted the compensation matrix, where necessary due to over- or under-compensation by the automatic algorithm. After adjustment of the compensation matrix, samples were concatenated and analyzed with the FlowJo UMAP plugin (v2.2). UMAP was run with the default settings (Euclidean distance function, nearest neighbors: 15 and minimum distance: 0.5). UMAP projections were obtained for concatenated cells from healthy donors (*n*=10), paucisymptomatic (*n*=8), pneumonia (*n*=13) and ARDS (*n*=10) patients. Total NK cells were identified as CD45^+^CD3^-^CD56^+^ cells. Among total NK cells, CD16 and CD57 were used to identify subsets defining maturation stages.

**Immune cell counts**

Absolute counts per µL of blood were determined with BD TBNK Trucount™ Tubes. Absolute counts for a particular cell population (A) were obtained by dividing the number of positive cell events (X) by the number of bead events (Y), and then multiplying by the BD Trucount bead concentration (N/V, where N = number of beads per test* and V = test volume). A = X/Y × N/V. The number of positive counts for each immune population was established with the following gating strategies: NK cells, CD45^+^CD3^-^CD19^-^CD16/CD56^+^ (CD16 and CD56 were detected using the same fluorochrome -PE conjugation); B cells, CD45^+^CD3^-^CD19^+^; CD4 T cells, CD45^+^ CD19^-^CD3^+^CD4^+^; CD8T cells CD45^+^ CD19^-^CD3^+^CD8^+^.

**NK cell cytotoxicity assay**

The functional activity of NK cells purified from the PBMCs of COVID-19 patients was assessed in a standard chromium release assay. Briefly, a preparation enriched in NK cells was obtained from PBMCs by magnetic negative selection, with a human NK cell isolation kit (MACS-Miltenyi Biotec), according to the manufacturer’s instructions. NK cells described as “resting” were left overnight in culture medium before the assay. NK cells were plated with tumor cells (K562 expressing HLA-E) loaded with chromium-51 (51Cr) (PerkinElmer) at an effector:target (E:T) cell ratio of 10:1 in U-bottomed 96-well plates (BD Falcon). Plates were incubated for 4 h at 37°C. After incubation, 50 μL of the culture supernatant was transferred to a LumaPlate (Perkin Elmer) coated with solid scintillator, which was then placed in a microplate scintillation counter (TopCount NXT, Perkin Elmer) to measure 51Cr release into the supernatant, which was correlated with target cell lysis. The following formula was used to calculate the percent specific lysis:

Specific lysis (%) = (experimental release – spontaneous release) / (maximal release − spontaneous release) ×100

The maximal release of 51Cr was determined by adding 2% Triton X-100 (Sigma-Aldrich) to the target cells, and spontaneous release was measured in medium alone without effector cells.

**Data analysis and statistics**

Statistical analyses and plots were done using GraphPad Prism (version 8.1.1). For the comparison of groups, *p*-values were obtained using two-sided Wilcoxon rank-sum tests. For paired expression analysis between blood and BALF or functional NK assays, *p*-values were obtained using two-sided Wilcoxon matched-pairs signed-rank tests. **p*<0.05; ***p*<0.01; ****p*<0.001;*****p*<0.0001. Boxplots represent the median and 25th to 75th percentiles, and the whiskers denote lowest and highest values.

**Explore COVID-19 group members**

The Explore COVID-19 IPH group: Joanna Farès, Laura Assante Miranda, William Baron, Nourhène Belaid, Clarisse Caillet, Flavien Caraguel, Sabrina Carpentier, Barbara Carrette, Florent Carrette, Fabien Chanuc, Rachel Courtois, Aurore Fenis, Marilyn Giordano, Mathilde Girard-Madoux, Marc Giraudon-Paoli, Nicolas Gourdin, Gwendoline Grondin, Franceline Guillot, Guillaume Habif, Solène Jaubert, Julie Lopez, Mélanie Le Van, Naouel Lovera, Marine Mansuy, Elodie Bonnet, Audrey Sansaloni, Annick Reboul, Emmanuel Mitry, Camille Nekkar-Constant, Valentine Péri, Romain Remark, Paul Ricaut, Léa Simon, Jean-Baptiste Vallier and Marie Vétizou. All members of the Explore COVID-19 group are employees of Innate Pharma.

The Explore COVID-19 Medical group: Sophie Ugolini, Marion Etiennot, Justine Galluso, Christelle Piperoglou, Luc Lyonnet, Jean-Marie Forel, Laurent Papazian, Lionel Velly, Baptiste André, Antoine Briantais, Benoit Faucher, Estelle Jean, Julie Seguier, Veronique Veit, Jean-Robert Harlé, Boris Pastorino, Bruno Coutard, Clémence Delteil, Laurent Daniel, Pierre Yves Cordier, Erwan Le Dault, Christophe Guervilly, Pierre Simeone, Marc Gainnier, Mikael Ebbo, Nicolas Schleinitz, Nassima Chouaki Benmansour, Jean-Paul Boudsocq, Axelle Clerc, Emmanuel Delmond, Pierre-Olivier Vidal and Hélène Savin.
